# Idiopathic and radiation-induced myxofibrosarcoma in the head and neck—case report and literature review

**DOI:** 10.1186/s41016-021-00267-9

**Published:** 2021-11-26

**Authors:** Bin Zhang, Miao Bai, Runfa Tian, Shuyu Hao

**Affiliations:** 1 Department of Neurosurgery, Beijing Tian Tan Hospital, Capital Medical University, No.119 South Fourth Ring West Road, Fengtai District, Beijing, 100070 People’s Republic of China; 2 Department of Neurology, Tang Du Hospital, Air Force Medical University, Xi’an, China

**Keywords:** Myxofibrosarcoma, Head and neck, Planned surgery, Gross total resection, Bin Zhang and Miao Bai are co-first authors

## Abstract

**Background:**

Myxofibrosarcoma (MFS), especially radiation-Induced MFS (RIMFS) in the head and neck, is an extremely rare malignant fibroblastic tumor. The diagnosis and treatment of MFS remain great challenges. In the present study, we presented one case of RIMFS. Combined with previous literature, the clinical features, essentials of diagnosis, and treatment modalities of MFS in the head and neck were reviewed to better understand this rare entity.

**Case presentation:**

We reported a case of RIMFS under the left occipital scalp in a 20-year-old girl with a history of medulloblastoma surgery and radiotherapy in 2006. A total tumor resection was performed with preservation of the overlying scalp the underlying bone, and no adjuvant therapy was administered after the first operation. The postoperative pathological diagnosis was high-grade MFS. The tumor relapsed 6 months later, and then, a planned extensive resection with negative surgical margins was carried out, followed by radiotherapy. No relapse occurred in a 12-month postoperative follow-up.

**Conclusions:**

Planned gross total resection (GTR) with negative margins is the reasonable choice and footstone of other treatments for MFS. Ill-defined infiltrated borders and the complicated structures make it a great trouble to achieve total resection of MFS in the head and neck, so adjuvant radiotherapy and chemotherapy seem more necessary for these lesions.

## Background

Myxofibrosarcoma (MFS) is a rare soft tissue sarcoma that can arise sporadically or be induced by radiation, representing approximately 5% of all sarcomas. MFS is one of the common soft tissue tumors in the extremities of elderly patients, which also occurs in the trunk (12%), retroperitoneum, or mediastinum (8%) [1]. In contrast, MFS, especially radiation-induced MFS (RIMFS) in the head and neck, is extremely rare.

MFS normally manifests as a painless and slow-growing dermal or subcutaneous mass. Clinically, it is characterized by tumor progression with increased metastasis rate after local recurrence [2, 3]. MRI is the most common pre-operative diagnostic modality. Histological grading of primary MFS is determined according to the updated French Federation of Cancer Centers (FNCLCC) scheme [4]. Due to the high rate of recurrence, planned gross total resection (GTR) with clear margins is essential and adjuvant treatment involving radiotherapy and chemotherapy is advised. However, due to ill-defined infiltrated borders and complex anatomical structures in the head and neck region, it is technically harder to achieve gross total resection [5]. Therefore, radiotherapy as well as chemotherapy looks more necessary for MFS in the head and neck than in the extremity.

To the best of our knowledge, only 28 cases have been reported in the head and neck so far, and 3 of them were induced by radiation (Table 1) [6–30]. Our case is the first case of scalp MFS following radiation exposure in a young female. Given its relatively recent recognition and the low incidence, only a single case or very small series have been reported, there are no randomized trials to guide treatment protocols. Without standard treatment protocol, it appears challenging to precisely predict prognosis for primary MFS by evaluating clinicopathological factors. Herein, we reported a case of radiation-induced scalp MFS in a 20-year-old girl with a history of medulloblastoma surgery and radiotherapy in 2006. Based on case report and literature review, we discussed clinical and histopathological features, treatment strategies, and prognostic factors of MFS in the head and neck, in order to contribute to a better understanding of this potentially fatal malignancy.

**Table 1:** Summary of reported cases of myxofibrosarcoma in head and neck

Case number	Author/year	Sex/age (year)	Radiation-induced (yes/no)	Location	Image	Biopsy (yes/no)	Treatment	Tumor margin	LR (yes/no)	Metastasis (yes/no)	Follow-up (month)
1	Lam PK et al., 2002^6^	M/55	No	Sphenoid sinus	CT, MRI	Yes	S	NE	No	No	8
2	Udaka T et al., 2002^7^	M/55	No	Neck	CT, MRI	No	S	NE	No	No	27
3	Nishimura G et al., 2006^8^	M/69	No	Hypopharynx	CT, MRI	Yes	S	PO	No	No	16
4	Kuo J et al., 2007^9^	M/28	Yes	Brain	CT, MRI	No	S + RT	N/A	N/A	N/A	N/A
5	Wang M et al., 2008^10^	F/63	No	Orbit	CT, MRI	No	S	PO	Yes	No	2
6	Enomoto K et al., 2008^11^	M/68	Yes	Sphenoid sinus	CT,PET	N/A	N/A	N/A	N/A	N/A	N/A
7	Gugatschka M et al.,2010^12^	M/79	No	Hypopharynx	Endoscopy, CT	No	S	NE	No	No	N/A
8	Li X et al., 2010^13^	F/37	No	Parotid	CT	No	S + RT	NE	No	No	8
9	Zhang Q et al., 2010^14^	F/27	No	Orbit	MRI	Yes	S + RT + C	NE	No	No	6
10	Buccoliero AM et al., 2011^15^	M/9	No	Brain	CT, MRI	No	S + RT + C	PO	Yes	No	15
11	Srinivasan B et al., 2011^16^	F78	No	Parotid	MRI	Yes	S + RT + C	PO	No	No	18
12	Norval EJG et al., 2011^17^	M69	No	Maxillary sinus	CT, MRI	Yes	RT + C	N/A	N/A	N/A	12
13	Gire J et al., 2011^18^	M/17	No	Orbit	CT,MRI	No	S	PO	No	No	24
14	Qiubei Z et al., 2012^19^	M42	No	Hypopharynx	CT	Yes	S	NE	No	No	36
15	Nakahara S et al., 2012^20^	M52	No	Maxillary sinus	MRI, Fdg-PET	Yes	S + RT	NE	No	No	17
16	Wernhart S et al., 2013^21^	M73	No	Brain	MRI	No	S + RT + C	N/A	N/A	Yes	2
17	Cante D et al., 2013^22^	M66	no	Maxillary sinus	CT, MRI	Yes	RT + C	N/A	N/A	Yes	18
18	Majumdar K et al., 2013^23^	F21	No	Brain	CT,MRI	No	S + RT	PO	Yes	No	30
19	Darouassi Y et al., 2014^24^	F74	No	Thyroid	CT	No	S + RT + C	N/A	Yes	No	N/A
20	Dell'Aversana OG et al., 2014^25^	M35	No	Maxillary sinus	CT, MRI	Yes	RT	N/A	No	No	27
21	Shimoda H et al., 2016^26^	M/67	No	Pterygopalatine fossa	CT	Yes	S + RT	PO	Yes	No	32
22	Costa DA et al., 2016^27^	M10	No	Brain	CT, MRI	N/A	S + RT	PO	Yes	Yes	N/A
23	Wong A et al., 2017^28^	F61	No	Maxillary sinus	CT, MRI	Yes	S + RT	N/A	N/A	N/A	N/A
24	Quimby A et al., 2017^29^	F/72	Yes	Brain, maxillary sinus, lung	CT, MRI	Yes	S + RT	PO	Yes	Yes	N/A
25	Tjarks BJ et al., 2018^30^	F/90	No	Scalp	N/A	Yes	S	N/A	Yes	Yes	N/A
26		M/65	No	Scalp	N/A	Yes	S	N/A	Yes	Yes	N/A
27		M/87	No	Scalp	N/A	No	S	N/A	N/A	N/A	N/A
28		M/70	No	Scalp	N/A	No	S	N/A	N/A	N/A	N/A
29	Present case	F20	Yes	Scalp	CT, MRI	No	S + RT	PO	YES	NO	18

Abbreviations: *C*, chemotherapy; *F*, female; *LR*, local recurrence; *M*, male; *NE*, negative; *PO*, positive; *RT*, radiotherapy; *S*, surgery

## Case presentation

In August 2016, a 20-year-old Chinese girl presented to our hospital with a 4-month history of finding a rapidly progressive palpable scalp swelling. Ten years ago, she was diagnosed with medulloblastoma in the fourth ventricle without leptomeningeal dissemination. Histopathologic examination revealed a classic type (WHO grade IV). Then, she received V-P shunt and surgical resection, as well as adjuvant concurrent chemoradiation (craniospinal irradiation 23.4 Gy, posterior fossa irradiation 55 Gy, and adjuvant chemotherapy).

**Fig. 1:**
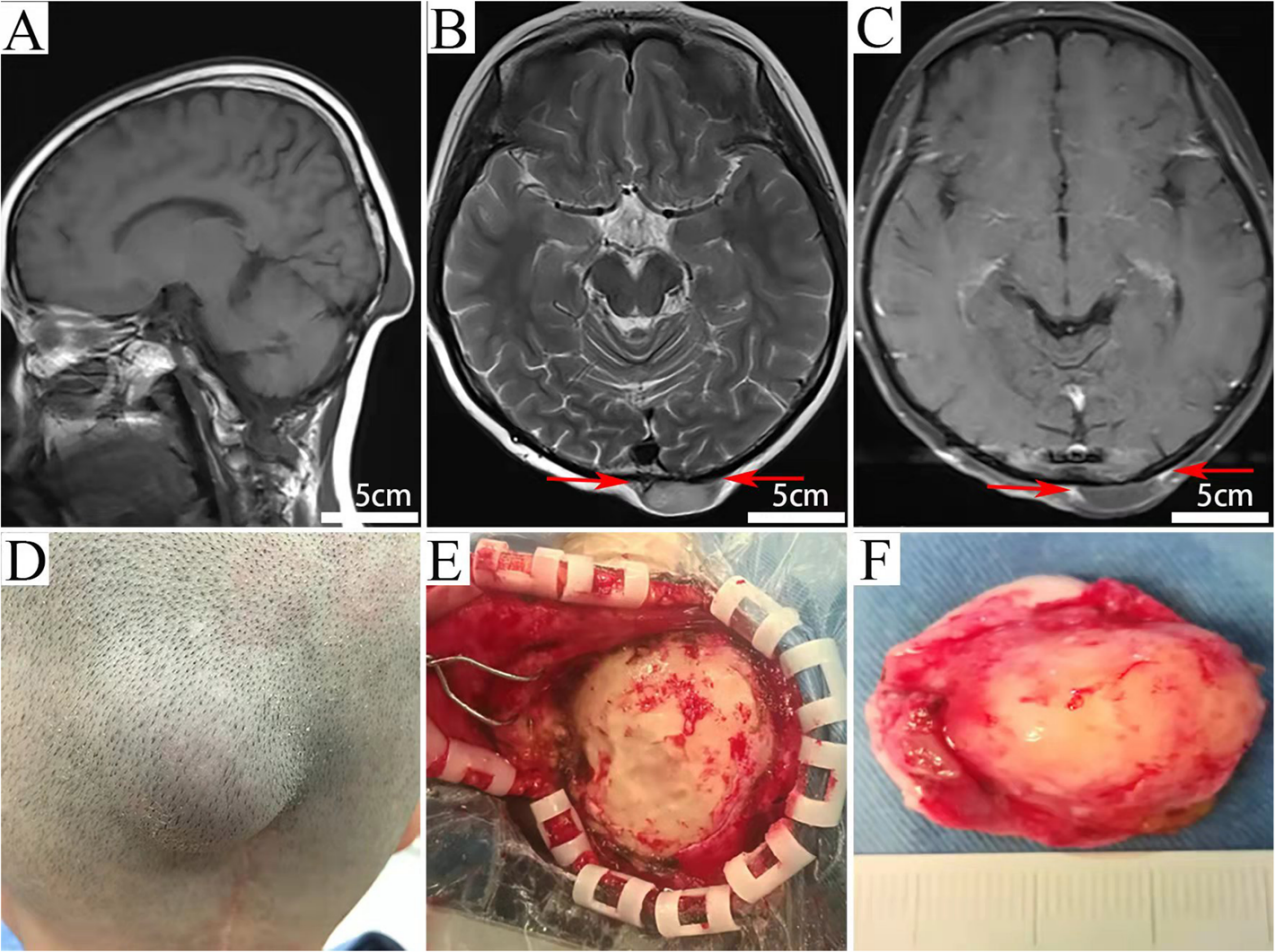
T1-weighted image (**A**), T2-weighted image (**B**), and contrast-enhanced MRI scans (**C**) reveal a lesion with well-defined borders under the left occipital scalp. It exhibits hypointensity on the T1-W sequence image (**A**), slightly hyperintensity on the T2-W axial image (**B**) and mild peripheral enhancement after contrast administration (**C**). “Tail sign” is found on T2-W axial image (**B**, red arrows), and is more obvious in the Post-contrast images (**C**, red arrows); Intraoperative photographs show the skull was compressed and deformed by the tumor (**E**). The tumor is grayish and about 35 × 25 cm in size (**F**)

Physical examination revealed that the lesion was under the left occipital scalp beside the up end of the incision, painless, firm in consistency, and immobile. Neurological examination was unremarkable. On MR imaging, the lesion exhibited a well-demarcated hypointense mass on T1­W sequences, slightly hyperintense on T2­W sequences, and peripheral enhancement with obvious “tail sigh” on contrast administration (Fig. 1). No biopsy was performed before the first operation. A gross total resection was carried out. Intra-operatively, the tumor was grayish, firm, well-demarcated with insufficient blood supply. The size of the tumor was approximately 35 × 25 cm. The mass was excised with preservation of the overlying scalp and the underlying bone (Fig. 1). Post-operative MRI image showed no residual tumor and no adjuvant therapy was administered. The girl made an uneventful recovery and was discharged on the six post-operative days.

**Fig. 2:**
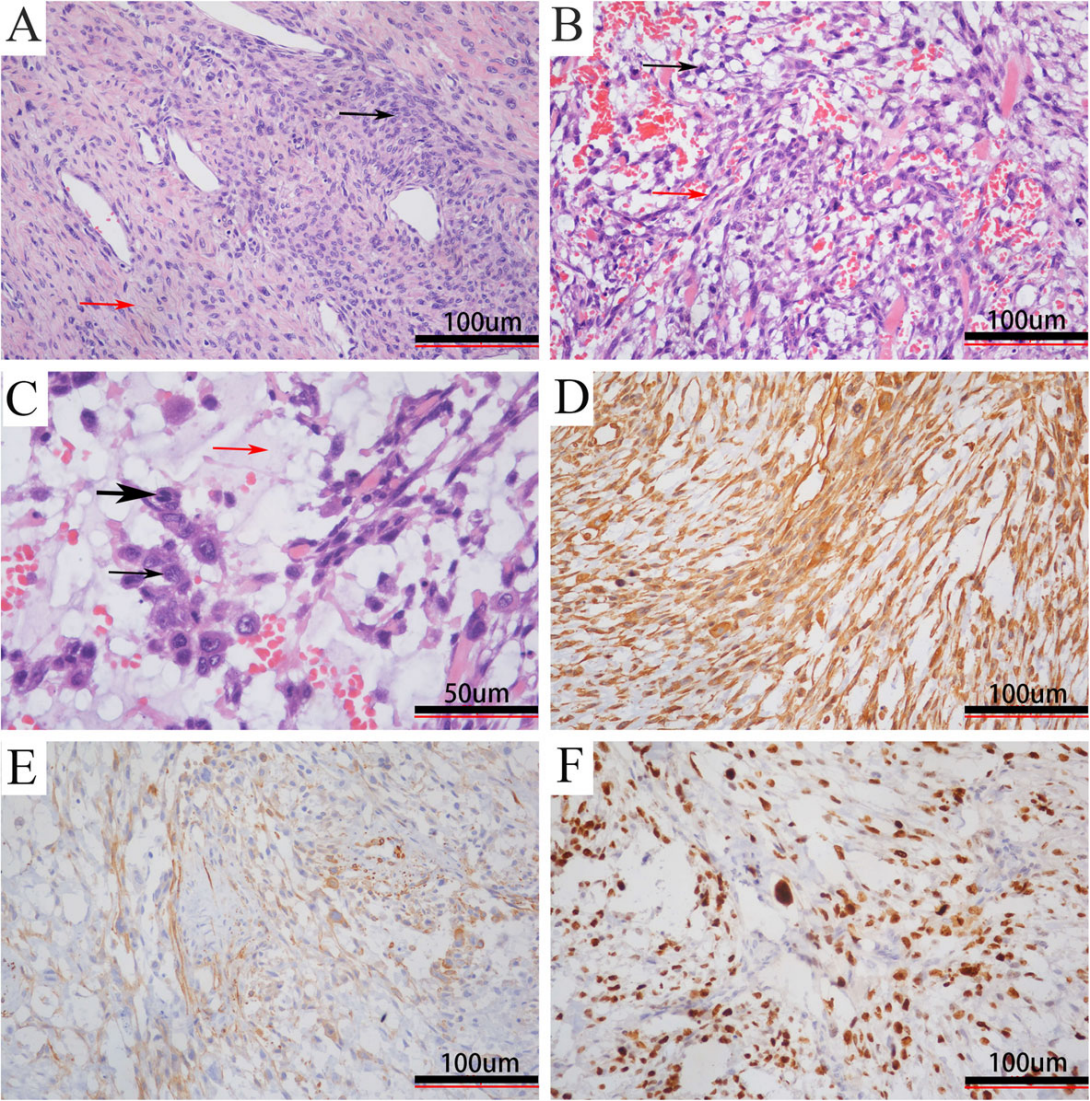
Histopathological examination. Hematoxylin and eosin [H&E] showing (**A**, × 100) alternating hypocellular (red arrow) and hypercellular (black arrow) areas, (**B**, × 200) spindle (red arrow) and stellate cells (black arrow), (**C**, × 200) tumor cells with pleomorphic (black arrow), and mitotic (thick black arrow) nuclei in the prominent myxoid matrix (red arrow); immunohistochemistry demonstrating positive staining for (**D**, × 200) vimentin and (**E**, × 200) SMA with a high (**F**, × 200) Ki-67 index (more than 50% of tumor cells)

Histopathologic examination showed that the tumor was composed of a myxoid matrix, curvilinear capillaries, and solid sheets of spindled cells, which were arranged in fascicles and sheets with a multinodular growth pattern and were supported by delicate, elongated, and curvilinear vasculature. There were more than 20 mitotic figures per high power field and necrosis was found in many areas. Immunohistochemical staining was positive for vimentin and SMA and negative for S-100, EMA, CD34, and myogenin. The Ki67 index is 50% (Fig. 2). The tumor was diagnosed as a high-grade MFS. Pathology was reviewed by experts in Peking Union Medical College Hospital.

Unfortunately, the tumor recurred in situ 6 months later. But this time, an extensive resection together with the overlying scalp and the underlying bone was performed, followed by cranioplasty and skin flap transplantation. The surgical margin was about 2 cm and was microscopically free of tumor confirmed by intra-operative frozen pathological examination. After surgery, the patient received radiotherapy (total dose, 60 Gy). No relapse occurred in a 12-month postoperative follow-up.

## Discussion

MFS was first described in 1977 [31], the high-grade end of MFS was considered as a part of the myxoid variant of Malignant fibrous histiocytoma (MFH), while the poorly recognized low-grade variant was construed as a part of the morphological continuum of MFS by Mentzel et al. until the late 1990s [1]. Given the use of modern methods including immunohistochemistry and molecular studies, MFS was proven to be not of true histiocytic origin but of fibroblastic origin and was defined as a distinct type of fibroblastic sarcoma by the WHO in 2002.^32^

MFS usually develops in proximal extremities of older people with a mean age of 65 years, men are usually affected slightly more often than women [32]. MFS in the head and neck is extremely rare, representing approximately 3% of MFS. To the best of our knowledge, only 28 cases have been reported so far, including brain (5, 17.9%), maxillary sinus (5, 17.9%), scalp (4, 14.2%), orbit (3, 10.7%), hypopharynx (3, 10.7%), sphenoid sinus (2, 7.2%), parotid (2, 7.2%), infratemporal space (2, 7.2%), thyroid gland (1, 3.5%), and multiple lesions (1, 3.5%) (Table 1). The psaranasal sinus appears to be the most frequent site, especially the maxillary sinus, followed by the brain. Similar to MFS in other regions, MFS in the head and neck mainly affects the older male patients (M/F = 19:11). Although the age range is broad, most patients are in their fifth to seventh decades of life, with a mean age of 40.9 years. In contrast, the onset age of RIMFS is associated with the time of receiving radiotherapy.

MFS in the extremities usually presents as a slowly enlarging and painless mass [33]. Due to the complexity of the anatomical structure, MFS in the head and neck illustrates a wide variety of manifestations ranging from an exophytic mass to focal neurological deficiency and symptoms of intracranial hypertension, such as headache and vomiting [6–30]. In our case, the tumor was a superficial type which presented as a rapidly progressive enlarging and painless mass. Clinically, MFS is characterized by its unusual infiltrative growth pattern, significant propensity for local recurrence, and tumor progression with increased metastasis rate after local recurrence.

Radiation-induced sarcomas (RIS) are increasingly seen in long-term survivors of head and neck tumors, with an estimated risk of up to 0.3%. Common histologic subtypes of RIS parallel their idiopathic counterparts and mainly include osteosarcoma, chondrosarcoma, malignant fibrous histiocytoma, and fibrosarcoma [34]. Radiation-induced MFS is very rare; only 3 cases have been reported until now. The diagnosis of RIS requires the following criteria [35]: (1) history of radiotherapy; (2) asymptomatic latency period of several years (conventionally, > 4 years); (3) occurrence of sarcoma within a previously irradiated field; and (4) histological confirmation of the sarcomatous nature of the post-irradiated lesion. Our case met all the criteria for RIS, including the development of myxofibrosarcoma within the radiation field, a 10-year latent period, and a different histopathological type.

MRI is the most common diagnostic modality for MFS. Computed tomography (CT) is also effective, especially for those located near the air and bone. MFS has a low density on CT, a low-to-intermediate signal on T1-weighted MRI, and a high signal on T2-weighted MRI. MFS often shows abnormal signal infiltration along the facial plan on MRI that corresponds to an infiltrative growth pattern histologically, named “tail sign.” Post-contrast images can better display “tail sign” than T2-weighted images [36, 37]. Thus, in order to define the boundaries of the tumor before operation, high-quality T1- and T2-weighted MRI with pre-and post-gadolinium imaging are necessary. However, due to a lack of typical MRI features, it is a great challenge to differentiate MFS from other tumors especially meningiomas which have iso- to hyperdense on CT, iso- to hypointense on T1 and T2, homogeneous enhancement, and the typical “tail sign.”

The definitive diagnosis of MFS depends on pathological examination. Histologically, a series of general parameters must be present such as spindle-shaped cells, elongated and pleomorphic nuclei, and an abundance of curvilinear vessels with thin walls and a myxoid matrix [38]. Low-grade MFSs are associated with a small amount of cells, a large amount of myxoid tissue, low mitotic activity, and no necrosis, while high-grade MFSs present with a large population of cells, less myxoid matrix, multinucleated giant cells, increased mitotic index, and important areas of necrotic tissue; the intermediate-grade tumors lend particularities of the other two but in a smaller amount, without well-developed solid and necrotic areas or significant pleomorphic cells [38, 39]. Currently, no specific immunohistochemical markers are available to definitely diagnose MFS. However, positive for vimentin, CD-34, and negative for S-100 protein, muscle-specific actin, desmin, and myogenin can support the diagnosis. In addition, Ki-67 reflects the tumor aggression when it is intensely expressed, and high expression of minichromosome maintenance protein 2 may be correlated with a short-term recurrence.^39^

Like other sarcomas, GTR (including nerves, vessels, and any involved bone) with negative margins remains the primary treatment for MFS [40]. In order to fulfill a total resection, a planned operation based on biopsy and a high-quality MRI imaging is necessary. Biopsy is necessary to orientate the diagnosis or even establish the type of soft tissue sarcoma. Unfortunately, in many cases, the actual tumor boundaries were usually underestimated on MRI due to infiltrative growth along the facial planes. Thus, an extended resection is necessary for these individuals, although the extent of the resection remains controversial, various surgical margins from 1 to 5 cm have been reported previously [40–48]. In order to confirm that the surgical margin was microscopically free of tumor, intraoperative frozen section and postoperative histological examination are recommended. Merck et al. reported that the local recurrence rate was up to 33% in MFS patients who undergo primary unplanned resection, in comparison to 17% for primary wide resection because of the unusual infiltrative growth of MFS [49]. However, it is more technically difficult to achieve radical resection in the head and neck region, especially in the deep area. In the reviewed 28 cases, only 7 cases were reported to be totally resected with negative margins (Table 1). The total resection rate is far more lower than that in other parts of the body. For these patients, additional treatments such as radiotherapy or chemotherapy are helpful. Previous studies showed that radiotherapy and chemotherapy significantly reduce the local recurrence of sarcoma [50, 51]. Unfortunately, the role of adjuvant radiotherapy and chemotherapy in the treatment of MFS is less clear due to the rarity of this tumor. Only several small studies reported the efficacy of chemotherapy in MFS [51, 52]. Additionally, the sensitivity of RIMFS to radiotherapy remains to be proven since they are induced by radiation.

MFS is a locally aggressive tumor that has a propensity for local recurrence (LR). Even after complete resection, the risk of recurrence is still high, ranging from 16 to 57% (Table 2). In contrast, the metastatic rate of MFS is relatively low, between 20 and 25%; the most common site is the lung, followed by the pleura, lymph nodes, and bones [40–48]. LR is more common for MFS in the head and neck. In the reviewed 28 cases, the LR rate was 43% (9/21), and all the RIS cases developed tumor relapse. But only 6 (25%, 6/24) cases developed tumor metastasis. Additionally, the prognosis of patients with RIS is generally worse than that with primary sarcomas of a similar stage [34]. Due to a small sample size, varying diagnostic and grading criteria, and obscure definition of wide resection, the prognostic parameters for MFS are still controversial. Despite controversies, in most studies, margin status is the most important predictor of LR; wide resection and negative margin are positively related to low LR [40–48]. Therefore, margin-negative surgical resection is the cornerstone of treatment for MFS.

**Table 2.** Literature review of previous studies about MFS

Author/year	No. of cases	Sex (M/F)	Age (year)	Treatment (no.)		Tumor margin status (no.)		LR (%)	Metastasis (%)
				S	RT	NE	PO		
Ghazala CG et al., 2016^33^	50	35/15	68.4 (median)	49	37	21	28	14	28
Daniels J et al., 2014^40^	30	13/17	65.8 (mean)	30	23	N/A	N/A	26.7	5
Look Hong NJ et al., 2013^41^	69	38/31	62 (median)	69	53	14	55	16	16
Riouallon G et al., 2013^42^	21	10/11	67 (mean)	21	21	17	4	57	9.5
Kikuta K et al., 2013^43^	100	61/39	64 (mean)	100	16	28	72	21	11
Dewan V et al., 2012^44^	172	N/A	67 (mean)	166	N/A	45	127	17	20
Haglund KE et al., 2012^45^	36	21/15	72.5 (median)	36	28	9	27	31	17
Sanfilippo R et al., 2011^46^	158	89/69	64 (mean)	158	81	28	130	18.2	14.6
Lin C et al., 2006^47^	70	38/32	64 (median)	61	28	26	43	44	23
Huang H et al., 2004^48^	49	26/23	60.5 (median)	49	9	19	28	57	16.3
Mentzel T et al., 1996^1^	75	N/A	66 (median)	74	13	N/A	N/A	54	22

Abbreviations: *F*, female; *LR*, local recurrence; *M*, male; *NE*, negative; *PO*, positive; *RT*, radiotherapy; *S*, surgery

## Conclusions

MFS is a locally aggressive tumor that has a propensity for local recurrence. Effective education about MFS, high-quality MRI imaging, biopsy, correct early diagnosis, and planned and wide surgical excision with negative margins are mandatory in order to provide the best results for MFS patients. Unfortunately, complex anatomical structures make MFS in the head and neck a great “challenge” to obtain a wide surgical margin. Therefore, in order to avoid local recurrence and distant metastasis, combined surgery and adjuvant chemoradiotherapy are recommended for MFS in this region. Further randomized double-blind controlled clinical trials are needed to confirm the efficacy of combined chemoradiotherapy for MSF in the head and neck.

## Data Availability

Not applicable.

## Abbreviations

CT: Computed tomography
GTR: Gross total resection
LR: Local recurrence
MFS: Myxofibrosarcoma
MRI: Magnetic resonance imaging
RIS: Radiation-induced sarcoma
MFH: Malignant fibrous histiocytoma

## References

[1] Mentzel T, Calonje E, Wadden C, Camplejohn RS, Beham A, Smith MA, Fletcher CDM (1996). Myxofibrosarcoma: clinicopathologic analysis of 75 cases with emphasis on the low-grade variant. Am J Surg Pathol..

[2] Huang H, Lal P, Qin J, Brennan MF, Antonescu CR (2004). Low-grade myxofibrosarcoma: a clinicopathologic analysis of 49 cases treated at a single institution with simultaneous assessment of the efficacy of 3-tier and 4-tier grading systems. Human Pathology..

[3] Willems SM, Debiec-Rychter M, Szuhai K, Hogendoorn PCW, Sciot R (2006). Local recurrence of myxofibrosarcoma is associated with increase in tumour grade and cytogenetic aberrations, suggesting a multistep tumour progression model. Modern Pathology..

[4] Neuville A, Chibon F, Coindre JM (2014). Grading of soft tissue sarcomas: from histological to molecular assessment. Pathology..

[5] Ghazala CG, Agni NR, Ragbir M, Dildey P, Lee D, Rankin KS, Beckingsale TB, Gerrand CH (2016). Myxofibrosarcoma of the extremity and trunk: a multidisciplinary approach leads to good local rates of LOCAL control. Bone Joint..

[6] Lam PK, Trendell-Smith N, Li JH, Fan YW, Yuen AP (2002). Myxofibrosarcoma of the sphenoid sinus. J Laryngol Otol..

[7] Udaka T, Yamamoto H, Shiomori T, Fujimura T, Suzuki H (2006). Myxofibrosarcoma of the neck. The Journal of Laryngology & Otology..

[8] Nishimura G, Sano D, Hanashi M, Yamanaka S, Tanigaki Y, Taguchi T, Horiuchi C, Matsuda H, Mikami Y, Tsukuda M (2006). Myxofibrosarcoma of the hypopharynx. Auris Nasus Larynx..

[9] Kuo J, Chio C, Wang C, Chu Y, Lin K, Chuang S (2007). Radiation-induced intra- and extra-cranial high-grade myxofibrosarcoma with tumor bleeding. Journal of Clinical Neuroscience..

[10] Wang M, Khurana RN, Parikh JG, Hidayat AA, Rao NA (2008). Myxofibrosarcoma of the orbit: an underrecognized entity?. Ophthalmology..

[11] Enomoto K, Inohara H, Hamada K, Tamura M, Tomita Y, Kubo T, Hatazawa J (2008). FDG PET imaging of myxofibrosarcoma on the sphenoid sinus. Clinical Nuclear Medicine..

[12] Gugatschka M, Beham A, Stammberger H, Schmid C, Friedrich G (2010). First case of a myxofibrosarcoma of the vocal folds: case report and review of the literature. Journal of Voice..

[13] Li X, Chen X, Shi ZH, Chen Y, Ye J, Qiao L, Qiu JH (2010). Primary myxofibrosarcoma of the parotid: case report. BMC Cancer..

[14] Zhang Q, Wojno TH, Yaffe BM, Grossniklaus HE (2010). Myxofibrosarcoma of the orbit: a clinicopathologic case report. Ophthalmic Plastic & Reconstructive Surgery..

[15] Buccoliero AM, Castiglione F, Garbini F, Rossi Degl’Innocenti D, Moncini D, Franchi A (2011). Primary cerebral myxofibrosarcoma: clinical, morphologic, immunohistochemical, molecular, and ultrastructural study of an infrequent tumor in an extraordinary localization. Journal of pediatric hematology/oncology..

[16] Srinivasan B, Ethunandan M, Hussain K, Ilankovan V (2011). Epitheloid myxofibrosarcoma of the parotid gland. Case reports in pathology.

[17] Norval EJG, Raubenheimer EJ (2011). Myxofibrosarcoma arising in the maxillary sinus: a case report with a review of the ultrastructural findings and differential diagnoses. Journal of Maxillofacial and Oral Surgery..

[18] Gire J, Weinbreck N, Labrousse F, Denis D, Adenis J, Robert P (2012). Myxofibrosarcoma of the orbit. Ophthalmic Plastic & Reconstructive Surgery..

[19] Qiubei Z, Cheng L, Yaping X, Shunzhang L, Jingping F (2012). Myxofibrosarcoma of the sinus piriformis: case report and literature review. World journal of surgical oncology..

[20] Nakahara S, Uemura H, Kurita T, Suzuki M, Fujii T, Tomita Y, Yoshino K (2012). A case of myxofibrosarcoma of the maxilla with difficulty in preoperative diagnosis. International Journal of Clinical Oncology..

[21] Wernhart S, Woernle CM, Neidert MC, Bode B, Rushing EJ, Studer G, Fuchs I, Regli L, Sürücü O (2013). A deeply seated brain metastasis from a primary myxofibrosarcoma: case report. Clinical Neurology and Neurosurgery..

[22] Cante D, Franco P, Sciacero P, Girelli GF, Borca VC, Pasquino M, Tofani S, Bombaci S, Migliaccio F, Marra A, Numico G, la Porta MR, Ricardi U (2013). Combined chemoradiation for head and neck region myxofibrosarcoma of the maxillary sinus. Tumori Journal..

[23] Majumdar K, Mandal S, Saran RK, Gupta R (2013). Recurrent intracranial myxofibrosarcoma presenting as an extensive fronto-parieto-occipital SOL: an unusual sarcoma of meningeal origin. Clinical Neurology and Neurosurgery..

[24] Darouassi Y, Attifi H, Zalagh M, Rharrassi I, Benariba F (2014). Myxofibrosarcoma of the thyroid gland. European Annals of Otorhinolaryngology, Head and Neck Diseases..

[25] Dell’Aversana OG, Iaconetta G, Abbate V, Piombino P, Romano A, Maglitto F (2014). Head and neck myxofibrosarcoma: a case report and review of the literature. J Med Case Rep..

[26] Shimoda H, Yonezawa K, Shinomiya H, Otsuki N, Hashikawa K, Sasaki R, Komura E, Nibu KI (2016). Modified partial maxillary swing approach for myxofibrosarcoma in pterygopalatine fossa. Head Neck..

[27] Costa DA, Barata P, Gouveia E (2016). Mafra M.

[28] Wong A, Chan WPR, Mirani NM, Eloy JA (2017). Myxofibrosarcoma of the maxillary sinus. Allergy Rhinol (Providence)..

[29] Quimby A, Estelle A, Gopinath A, Fernandes R (2017). Myxofibrosarcoma in head and neck: case report of unusually aggressive presentation. Journal of Oral and Maxillofacial Surgery..

[30] Tjarks BJ, Ko JS, Billings SD (2018). Myxofibrosarcoma of unusual sites. J Cutan Pathol..

[31] Weiss SW, Enzinger FM (1977). Myxoid variant of malignant fibrous histiocytoma. Cancer..

[32] Fletcher C, Unni K (2002). Mer tens F. Pathology and genetics of tumours of soft tissue and bone. 3rd edition.

[33] Ghazala CG, Agni NR, Ragbir M, Dildey P, Lee D, Rankin KS, Beckingsale TB, Gerrand CH (2016). Myxofibrosarcoma of the extremity and trunk: a multidisciplinary approach leads to good local rates of local control. Bone Joint J..

[34] Rosko AJ, Birkeland AC, Chinn SB, Shuman AG, Prince ME, Patel RM, McHugh JB, Spector ME (2017). Survival and margin status in head and neck radiation-induced sarcomas and de novo sarcomas. Otolaryngol Head Neck Surg..

[35] Dickson MA (2014). Systemic treatment options for radiation-associated sarcomas. Curr Treat Options Oncol..

[36] Yoo HJ, Hong SH, Kang Y, Choi J, Moon KC, Kim H (2014). MR imaging of myxofibrosarcoma and undifferentiated sarcoma with emphasis on tail sign; diagnostic and prognostic value. European Radiology..

[37] Lefkowitz RA, Landa J, Hwang S, Zabor EC, Moskowitz CS, Agaram NP, Panicek DM (2013). Myxofibrosarcoma: prevalence and diagnostic value of the “tail sign” on magnetic resonance imaging. Skeletal Radiology..

[38] Mansoor A, White CR (2003). Myxofibrosarcoma presenting in the skin: clinicopathological featur es and differential diagnosis with cutaneous myxoid neoplasms. Am J Dermatopathol..

[39] Wincewicz A, Lewitowicz P, Matykiewicz J, Głuszek S, Sulkowski S (2015). Intramuscular high-grade myxofibrosarcoma of left buttock of 66-year-old male patient – approach to systematic histopathological reporting. Rom J Morphol Embryol..

[40] Daniels J, Green CM, Freemont A, Paul A (2014). The management of myxofibrosarcoma - a ten-year experience in a single specialist centre. Acta orthopaedica Belgica..

[41] Look Hong NJ, Hornicek FJ, Raskin KA, Yoon SS, Szymonifka J, Yeap B, Chen YL, DeLaney TF, Nielsen GP, Mullen JT (2013). Prognostic factors and outcomes of patients with myxofibrosarcoma. Annals of Surgical Oncology..

[42] Riouallon G, Larousserie F, Pluot E, Anract P (2013). Superficial myxofibrosarcoma. Assessment of recurrence risk according to the surgical margin following resection. A series of 21 patients. Orthopaedics & Traumatology: Surgery & Research..

[43] Kikuta K, Kubota D, Yoshida A, Suzuki Y, Morioka H, Toyama Y, Kobayashi E, Nakatani F, Chuuman H, Kawai A (2013). An analysis of factors related to recurrence of myxofibrosarcoma. Japanese Journal of Clinical Oncology..

[44] Dewan V, Darbyshire A, Sumathi V, Jeys L, Grimer R (2012). Prognostic and survival factors in myxofibrosarcomas. Sarcoma..

[45] Haglund KE, Raut CP, Nascimento AF, Wang Q, George S, Baldini EH. Recurrence patterns and survival for patients with intermediate- and high-grade myxofibrosarcoma. International Journal of Radiation Oncology*Biology* Physics, 367. 2012;82:361. 10.1016/j.ijrobp.2010.08.042 20951504

[46] Sanfilippo R, Miceli R, Grosso F, Fiore M, Puma E, Pennacchioli E, Barisella M, Sangalli C, Mariani L, Casali PG, Gronchi A (2011). Myxofibrosarcoma: prognostic factors and survival in a series of patients treated at a single institution. Annals of Surgical Oncology..

[47] Lin C, Chou S, Li C, Tsai K, Chen W, Hsiung C (2006). Prognostic factors of myxofibrosarcomas: Implications of margin status, tumor necrosis, and mitotic rate on survival. Journal of Surgical Oncology..

[48] Huang H (2004). Low-grade myxofibrosarcoma: a clinicopathologic analysis of 49 cases treated at a single institution with simultaneous assessment of the efficacy of 3-tier and 4-tier grading systems. Human Pathology..

[49] Merck C, Angervall L, Kindblom LG, Odén A (1983). Myxofibrosarcoma. A malignant soft tissue tumor of fibroblastic-histiocytic origin. A clinicopathologic and prognostic study of 110 cases using multivariate analysis. Acta Pathol Microbiol Immunol Scand Suppl..

[50] Beane JD, Yang JC, White D, Steinberg SM, Rosenberg SA, Rudloff U (2014). Efficacy of adjuvant radiation therapy in the treatment of soft tissue sarcoma of the extremity: 20-year follow-up of a randomized prospective trial. Ann Surg Oncol..

[51] Pervaiz N, Colterjohn N, Farrokhyar F, Tozer R, Figueredo A, Ghert M (2008). A systematic meta-analysis of randomized controlled trials of adjuvant chemotherapy for localized resectable soft-tissue sarcoma. Cancer..

[52] Colia V, Fiore M, Provenzano S, Fumagalli E, Bertulli R, Morosi C (2017). Activity of anthracycline- and ifosfamide-based chemotherapy in a series of patients afected by advanced myxofbrosarcoma. Clin Sarcoma Res.

